# Agar Acts as Cathode Microskin to Extend the Cycling Life of Zn//α-MnO_2_ Batteries

**DOI:** 10.3390/ma14174895

**Published:** 2021-08-27

**Authors:** Linqing Zuo, Haodong Sun, Xinhai Yuan, Juan Wen, Xi Chen, Shiyu Zhou, Yuping Wu, Teunis van Ree

**Affiliations:** 1 China State Key Laboratory of Materials-Oriented Chemical Engineering, Institute of Advanced Materials (IAM), School of Energy Science and Engineering, Nanjing Tech University, Nanjing 210009, China; 201861122095@njtech.edu.cn (L.Z.); Sun-Haodong@njtech.edu.cn (H.S.); 15026765265@163.com (X.Y.); wenjuan@njtech.edu.cn (J.W.); chenxi@njtech.edu.cn (X.C.); 201961108012@njtech.edu.cn (S.Z.); 2 Department of Chemistry, University of Venda, Thohoyandou 0950, South Africa; Teuns.VanRee@univen.ac.za

**Keywords:** aqueous rechargeable battery, positive electrode, manganese dioxide, high discharge capacity

## Abstract

The Zn/MnO_2_ battery is a promising energy storage system, owing to its high energy density and low cost, but due to the dissolution of the cathode material, its cycle life is limited, which hinders its further development. Therefore, we introduced agar as a microskin for a MnO_2_ electrode to improve its cycle life and optimize other electrochemical properties. The results showed that the agar-coating layer improved the wettability of the electrode material, thereby promoting the diffusion rate of Zn^2+^ and reducing the interface impedance of the MnO_2_ electrode material. Therefore, the Zn/MnO_2_ battery exhibited outstanding rate performance. In addition, the agar-coating layer promoted the reversibility of the MnO_2_/Mn^2+^ reaction and acted as a colloidal physical barrier to prevent the dissolution of Mn^2+^, so that the Zn/MnO_2_ battery had a high specific capacity and exhibited excellent cycle stability.

## 1. Introduction

In recent years, the lithium-ion battery has permeated our lives and production and is used in both small electronic appliances and large mechanical equipment [1,2]. However, some issues such as long charging time and flammability of electrolyte solvents restrict its wide application, especially in large-scale energy storage systems (ESSs) that require high safety and reliability [3,4,5,6]. Therefore, some researchers shifted their attention to research on aqueous rechargeable batteries [7,8,9,10,11,12]. Among various aqueous battery systems, aqueous Zn-ion battery (ZIB) is very widely studied, owing to its high theoretical specific capacity (820 mAh g^−1^), low redox potential (−0.76 V vs. Standard hydrogen electrode, SHE), and low cost of Zn anodes [13,14]. Although it has these attractive merits, improving its cycle life is still a problem that needs to be solved urgently. As an important part of the ZIB, the cathode material is one of the keys to solving its cycling stability. Until now, candidate cathodes for ZIBs have been based mainly on vanadium-based materials, manganese-based materials, Prussian blue analogues, and other metal oxides or metal chalcogenides [13]. Among them, manganese-based materials, especially tunnel-type α-MnO_2_, are considered promising materials due to their low synthesis cost and high energy density [15]. However, MnO_2_ suffers from serious capacity decline in aqueous electrolytes, owing to the dissolution of active materials and structural transformation caused by the disproportionation reaction of Mn^3+^. The addition of MnSO_4_ to electrolytes can inhibit this side reaction to a certain extent, because additional Mn^2+^ influences the balance between Mn^2+^ dissolution and reoxidation [16]. To further improve the cycling performance of MnO_2_, a common strategy is surface coating by conductive carbon materials or polymer materials, which cannot only enhance the electrical conductivity of MnO_2_ and ease volume changes, but also prevent Mn dissolution [17]. However, the improvement of cycle life is limited due to the poor reversibility of the MnO_2_/Mn^2+^ redox reaction [18]. Recently, Chen’s research group used collagen hydrolysate (CH) as an electrode microskin (EMS) to adsorb/confine dissolved Mn^2+^ ions, resulting in the reversibility of the redox reaction of MnO_2_/Mn^2+^ during the charge and discharge processes, thereby improving cycle performance [19]. This is a simple and effective method to improve the cycle stability of the Zn/MnO_2_ battery, which is worthy of further promotion and in-depth research.

Agar is a polysaccharide extracted from seaweed and is widely used in the food industry, pharmaceutical industry, chemical industry, bioengineering, and many other fields. Agar used in food can significantly change the quality of food and improve the quality of food [20]. It can be used as a thickener, a coagulant, a suspending agent, an emulsifier, a preservative, and a stabilizer. In addition, agar is also used in the field of electrochemistry as an electrolyte additive to alleviate metal corrosion, with excellent results [21,22,23,24,25]. Due to the above properties and advantages, agar may be promising for use as a cathode microskin for the Zn/MnO_2_ battery.

Here, we report the use of agar as the EMS of an α-MnO_2_ electrode to improve the electrochemical performance of a Zn//α-MnO_2_ battery. The influences of different thicknesses of an agar coating layer on the wettability and resistance of the α-MnO_2_ electrode and the rate performance, specific capacity, and cycling performance of the Zn//α-MnO_2_ battery were explored.

## 2. Experimental Section

### 2.1. Material Synthesis

#### 2.1.1. Preparation of α-MnO_2_

α-MnO_2_ was prepared by a hydrothermal method. Typically, 1 g KMnO_4_ (Analytical Reagent, AR, Sinopharm Chemical Reagent Co. Ltd., Shanghai, China) and 0.4 g MnSO_4_ (AR, Sinopharm Chemical Reagent Co. Ltd., Shanghai, China) were dissolved in 50 mL and 22 mL deionized water, respectively. Then, the two solutions were mixed, and the resulting mixture was stirred for 10 min at room temperature to form a clear solution. Afterwards, the solution was transferred to a 100 mL autoclave (Nanjing Wanqing Chemical Glass Ware & Instrument Co. Ltd., Nanjing, China) and reacted at 160 °C for 12 h. After cooling to room temperature, the brownish black product was collected by vacuum filtration and washed with deionized water followed by absolute ethanol (≥99.7%, Wuxi Yasheng Chemical Co. Ltd., Wuxi, China). The final product was dried in an oven (DGG-9240A, Shanghai Senxin Experimental Instrument Co. Ltd., Shanghai, China) at 80 °C for 12 h before use.

#### 2.1.2. Modification of α-MnO_2_ Electrode by Agar Coating

To prepare the α-MnO_2_ electrode, α-MnO_2_, acetylene black (AR, Aladdin, Shanghai, China), and poly(vinylidene fluoride) (PVDF) (AR, Aladdin, Shanghai, China) were mixed with a mass ratio of 7:2:1 in an appropriate amount of in N-methyl-2-pyrrolidinone (AR, Macklin Biochemical Co. Ltd. Shanghai, China), and the mixture was stirred for 3 h. Then, the stirred slurry was coated on a stainless-steel foil (thickness: 0.01 mm, Dongguan Pingjie Metal Material Co., Ltd. Dongguan, China) and dried in a vacuum drying oven (DZG-6050, Shanghai Senxin Experimental Instrument Co. Ltd., Shanghai, China) at 80 °C for 12 h. Finally, the foil was cut into a disc with a diameter of 5 mm for use. The active substance loaded per disc was about 2 mg.

Agar solutions with agar mass fractions of 1% and 2.5% were obtained by mixing agar (AR, Macklin Biochemical Co. Ltd. Shanghai, China) with ultra-pure water at mass ratios of 1:99 and 2.5:97.5, respectively, and the mixtures were heated at 90 °C for 1 h.

Ten microliters of each agar solution were dripped onto the cut α-MnO_2_ electrodes, which were dried on a hot plate at 60 °C to prepare agar-modified α-MnO_2_ electrodes. The electrodes coated with 1% and 2.5% agar solutions were denoted as MnO_2_-1 and MnO_2_-2, respectively.

### 2.2. Material Characterization

The structures of the α-MnO_2_ were determined by X-ray diffractometer (XRD, Rigaku, Smartlab TM, Tokyo, Japan) (Cu Kα; λ = 1.5406 Å). The morphologies of the α-MnO_2_ and different thicknesses of the agar coating layers were observed using scanning electron microscopy (SEM; JEOL Ltd., JSM-7800F, Tokyo, Japan). For more detailed material analyses, transmission electron microscopy (TEM; JEOL Ltd., JEM-2100F, Tokyo, Japan) was used, Fourier Transform Infrared (FTIR) analyses were performed on a Vertex 70 FTIR spectrometer (Bruker Optik GmbH, Leipzig, Germany).

### 2.3. Electrochemical Measurements

Both cyclic voltammetry (CV) and electrochemical impedance spectroscopy (EIS) were measured using a Chenhua 660 E electrochemical workstation (CH Instruments Inc, Shanghai, China). The rate and cycling performances were tested on a LAND 2001A battery test system (Wuhan LAND Electronic Co. Ltd., Wuhan, China). A 0.8 mm commercial zinc foil (Kunshan Yizhongtian New Material Co. Ltd., Suzhou, China), a 2 M ZnSO_4_ + 0.5 M MnSO_4_ solution, and a glass fiber membrane (Whatman GF/A, Maidstone, England) were used as an anode, an electrolyte, and a diaphragm, respectively. A button battery-packaging machine (Shenzhen Kejing Star Technology Co. Ltd. Shenzhen, China) was used to package CR2025 button batteries. After standing for 1 h, the electrochemical test was performed.

## 3. Results and Discussion

### 3.1. Characterization of the Physical Properties of the Prepared α-MnO_2_

**Figure 1:**
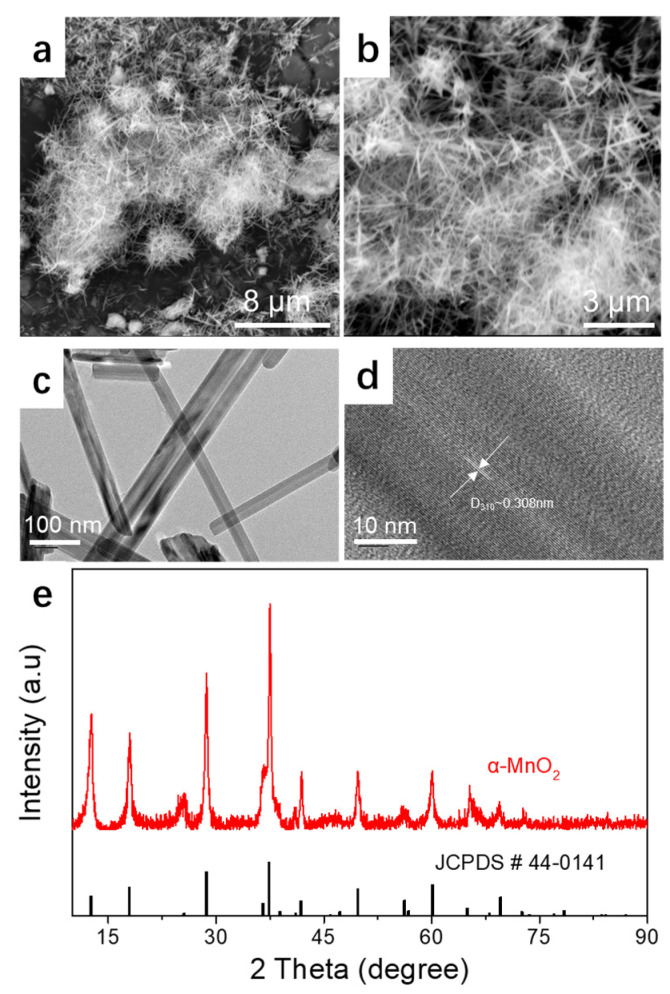
Physical properties of the prepared MnO_2_: (**a**,**b**) SEM images; (**c**,**d**) TEM images; (**e**) XRD patterns.

The physical properties of the prepared MnO_2_ are illustrated in Figure 1. The MnO_2_ nanorods, with a length of 2–3 μm and a thickness of 100–150 nm, can be clearly observed (Figure 1a–c). The TEM is shown in Figure 1d. A lattice spacing of 0.308 nm was found to match well with the (310) crystal planes of α-MnO_2_. The XRD patterns conformed to a pure α-MnO_2_ phase (JCPDS No. 44-0141) [26].

### 3.2. Characterization of the Agar-Modified Cathodes

**Figure 2:**
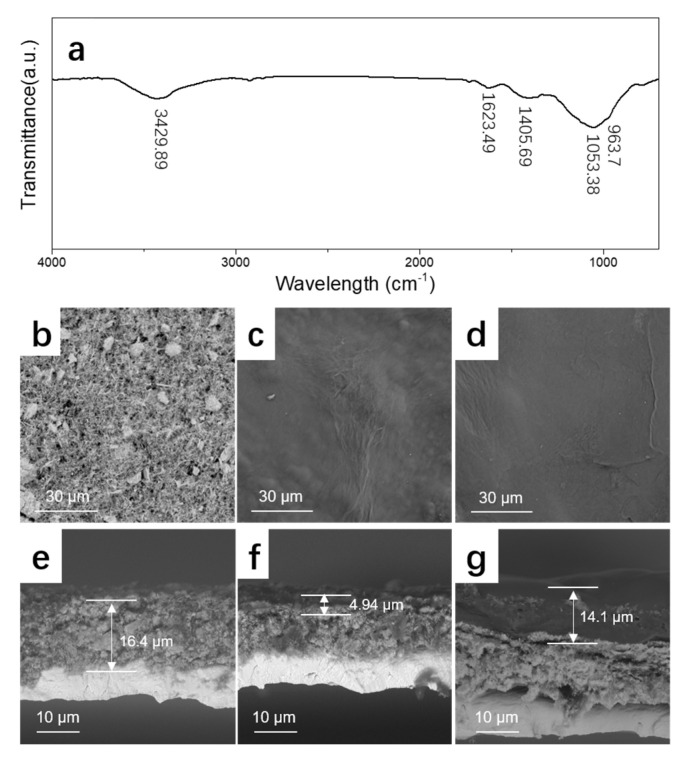
(**a**) FTIR spectrum of agar; SEM image of α-MnO_2_ (**b**,**e**), MnO_2_-1 (**c**,**f**), and MnO_2_-2 (**d**,**g**).

The FTIR spectrum of agar is shown in Figure 2a. The absorption band at 3429.89 cm^−1^ corresponds to the stretching vibration of the hydroxyl groups (O–H) in agar, which participate in the formation of intermolecular and intramolecular hydrogen bonds; the weak absorption band at 1623.49 cm^−1^ is allocated to the C=O stretching vibration; the band around 1405.69 cm^−1^ can be attributed to the sulfate ester group in agar; the absorption band at 1053.38 cm^−1^ corresponds to several C–O–H bending modes; the absorption band at 963.7 cm^−1^ is attributed to the C–O–C bending mode in 3,6-anhydrogalactose [22]. The FTIR spectra of ager coated MnO_2_ electrode is shown in Figure S1, which is consistent with that of pure agar. Figure 2b–d shows the top view of the SEM images of the α-MnO_2_ electrode and electrodes coated with agar. The surface of the α-MnO_2_ electrode was rough, while the agar-coated electrodes had smooth skins. The thicknesses of the microskins on the electrode surfaces coated with agar solutions of different concentrations were 4.94 μm and 14.1 μm for MnO_2_-1 and MnO_2_-2, respectively.

**Figure 3:**
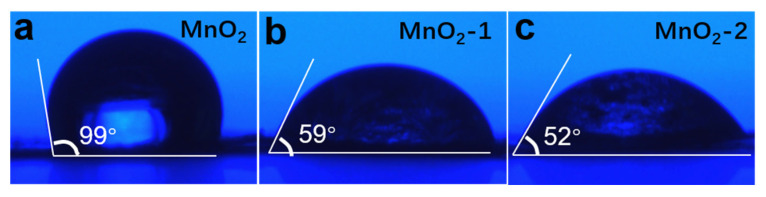
Contact angle photos of the electrolyte on α-MnO_2_ (**a**), MnO_2_-1 (**b**), and MnO_2_-2 (**c**).

It has been shown that electrode wettability is closely related to the capacity and cycle life of the battery. Generally speaking, improving the wettability of electrode materials can shorten the wetting time, make full use of the electrode capacity and provide better electrochemical performance [27,28]. The surface hydrophilicity tests results of the α-MnO_2_ electrode and electrodes coated with agar are shown in Figure 3. It can be seen that the contact angle was reduced from 99° to 52° with increasing amounts of agar on the cathode. This means that agar coating may increase the diffusion rate of Zn^2+^ on the MnO_2_ electrode surface, reduce the interface impedance and improve the rate performance.

### 3.3. Electrochemical Performance of Zn//α-MnO_2_ Batteries

**Figure 4:**
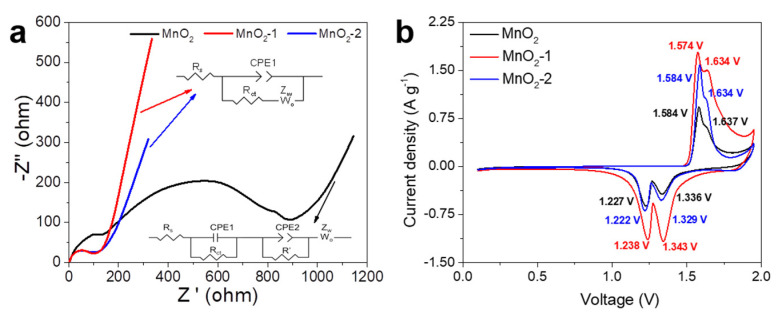
(**a**) Electrochemical impedance spectroscopy (EIS) spectra; and (**b**) cyclic voltammetry (CV) curves of the three batteries at a scan rate of 0.3 mV s^−1^.

Zn//α-MnO_2_ batteries were assembled using a 2 M ZnSO_4_ + 0.5 M MnSO_4_ aqueous solution as an electrolyte. In order to explore the effect of agar as a cathode microskin on the electrochemical performance of Zn//α-MnO_2_ batteries, its EIS spectrum was first measured. As shown in Figure 4a, the Zn/MnO_2_-1 battery showed the lowest charge transfer resistance in the high-frequency area, which may be attributed to the decrease of the cross-sectional impedance of the electrode due to the improvement of its wettability [29,30]. The charge transfer resistance of the Zn/MnO_2_-2 battery came second, because although the cathode had the best wettability, the agar microskin coating was thicker, which increased the interface resistance. The slope of the low-frequency parts was calculated, as shown in Figure S2. The slopes for the Zn/MnO_2_ battery, Zn/MnO_2_-1 battery, and Zn/MnO_2_-2 battery are 1.01, 2.78, and 1.54, respectively. The slope of the Zn/MnO_2_-1 battery was the highest, indicating that the diffusion rate of Zn^2+^ was the highest in the MnO_2_-1 electrode. In Figure 4b, the CV curves of the three batteries at a scan rate of 0.3 mV s^−1^ over the potential range of 0.1–1.95 V (vs. Zn^2+^/Zn) are shown. The CV curves of these three batteries had similar shapes, with two reduction peaks and two oxidation peaks, which can be attributed to the redox processes of Mn^4+^/Mn^3+^ caused by the intercalation of Zn^2+^ into the MnO_2_ host and the extraction of Zn^2+^ from the MnO_2_ host, respectively. Compared with the Zn/MnO_2_ battery and the Zn/MnO_2_-2 battery, the Zn/MnO_2_-1 battery had a higher peak current density, indicating that it had the highest electrochemical activity and capacity [31]. In addition, the Zn/MnO_2_-1 battery had the lowest polarization, meaning that it had the lowest interface impedance, which is consistent with the EIS test results.

**Figure 5:**
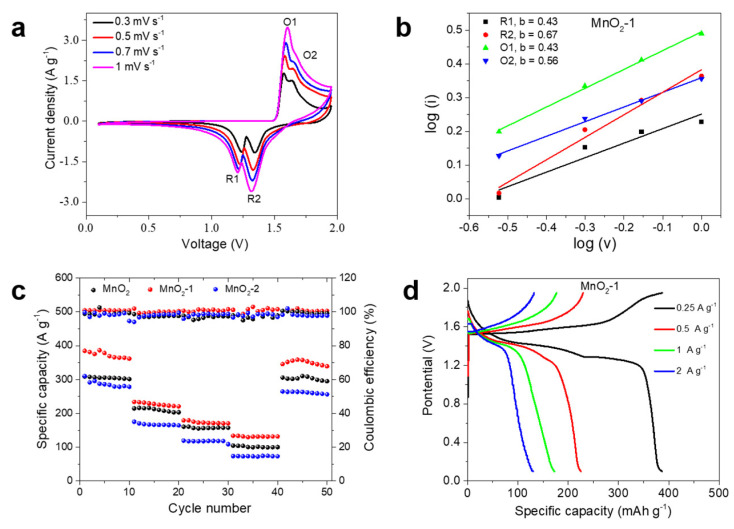
(**a**) CV curves of the Zn/MnO_2_-1 battery at various scan rates over the potential range of 0.1–1.95 V; (**b**) a linear fit of log(i) as a function of log(v); (**c**) rate performances of the three batteries; (**d**) charge–discharge curves of the Zn/MnO_2_-1 battery at different current densities.

The CV curves of the Zn/MnO_2_-1 battery at various scan rates from 0.3 to 1 mV s^−1^ are shown in Figure 5a. With the increasing scan rate, it can be seen clearly that the shapes of the CV curves are consistent, but the peaks in the CV gradually grow wider. According to the equation: log(i) = log(a) + blog(v), where the b values were calculated as 0.43 (R1), 0.67 (R2), 0.43 (O1), and 0.56 (O2) by fitting the linear relationship between log(i) and log(v) (Figure 5b). This clearly shows that the ion diffusion process and surface capacitive effects both contributed to the electrochemical kinetics of the MnO_2_-1. Figure 5c records the rate performances of the Zn/MnO_2_ battery, the Zn/MnO_2_-1 battery, and the Zn/MnO_2_-2 battery at various current densities. The results showed that the rate performance of the Zn/MnO_2_-1 battery was the best, followed by that of the Zn/MnO_2_-2 battery, while the Zn/MnO_2_ battery performed the worst. The Zn/MnO_2_-1 battery delivered a capacity of 384.7, 232.8, 179.3, and 133.3 mAh g^−1^ at current densities of 0.25, 0.5, 1.0, and 2 A g^−1^, respectively. The corresponding charge and discharge curves are shown in Figure 5d. It can be seen that when the current density was 0.25 A g^−1^, there were two plateaus on the charge–discharge curve, which are consistent with the CV curve. It is worth noting that as the current density increased, the second plateau gradually disappeared. This may be due to the slow diffusion of Zn^2+^ at high current density; its capacity contribution mainly comes from H^+^ insertion/extraction [32]. The specific capacity of the Zn/MnO_2_ battery at the corresponding current density was much lower than that of the Zn/MnO_2_-1 battery (Figure S3), which indicated that the agar microskin improved the reversibility of the MnO_2_/Mn^2+^ reaction [19]. The specific capacity of the Zn/MnO_2_-2 battery was higher than that of the Zn/MnO_2_ battery, but lower than that of the Zn/MnO_2_-2 battery. This is mainly because the agar microskin of the MnO_2_-2 electrode was thicker than that of the MnO_2_-1 electrode, which hindered the transmission speed of Zn^2+^ to a certain extent.

**Figure 6:**
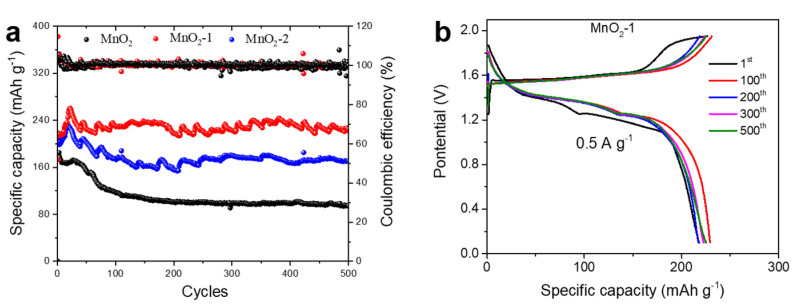
(**a**) Cycling performances of the three batteries at a current density of 0.5 A g^−1^ in the potential range between 0.1 and 1.95 V; (**b**) electrochemical charge–discharge profiles of MnO_2_-1.

The cycling performance was studied by continually charging and discharging at 0.5 A g^−1^. Figure 6a compares the cycling performance of the Zn//α-MnO_2_ batteries with that of the Zn/MnO_2_-1 battery exhibiting the best cycling performance with a capacity retention of 95.4% after 500 cycles and with the highest specific capacity, which was substantially higher than the values for most Zn/MnO_2_ batteries reported to date (Table 1). This superior performance can be attributed mostly to the agar coating. During the charge and discharge processes, agar acted as a colloidal physical barrier to inhibit the dissolution of Mn^2+^, hence improving the cycling performance of the Zn//α-MnO_2_ battery. Moreover, due to the improvement of the reversibility of the MnO_2_/Mn^2+^ reaction by agar coating, the specific capacities of the Zn/MnO_2_-1 battery and the Zn/MnO_2_-2 battery were higher than that of the Zn/MnO_2_ battery. However, due to the thick coating layer of MnO_2_-2, the migration rate of Zn^2+^ in this battery was hindered to a certain extent, and the interface impedance was increased, so that the specific capacity of the Zn/MnO_2_-2 battery was lower than that of the Zn/MnO_2_-1 battery. It should be noted that both the Zn/MnO_2_-1 battery and the Zn/MnO_2_-2 battery had an activation process during cycling and the specific capacity reached a maximum after about 20 cycles, after which the capacity remained relatively stable. The specific capacity of the Zn/MnO_2_ battery decayed rapidly over the first 100 cycles and then more slowly. After 500 cycles, the capacity retention rate was only 51.6%. The charge and discharge curves of the Zn/MnO_2_-1 battery over different cycles are shown in Figure 6b. The charge and discharge curves remained stable over 500 cycles, which also means that the structure of the MnO_2_-1 electrode remained stable, indicating that the agar coating inhibited the dissolution of Mn^2+^ and maintained the structural stability of the α-MnO_2_. Compared with that of the Zn/MnO_2_ battery, the cycling stability of the Zn/MnO_2_-2 battery was also greatly improved (Figure S4b). In contrast, the charge–discharge curves of the Zn/MnO_2_ battery varied greatly during different charge–discharge cycles (Figure S4a). After 100 cycles, the second discharge plateau disappeared, which means that the structure of the α-MnO_2_ was destroyed.

**Figure 7:**
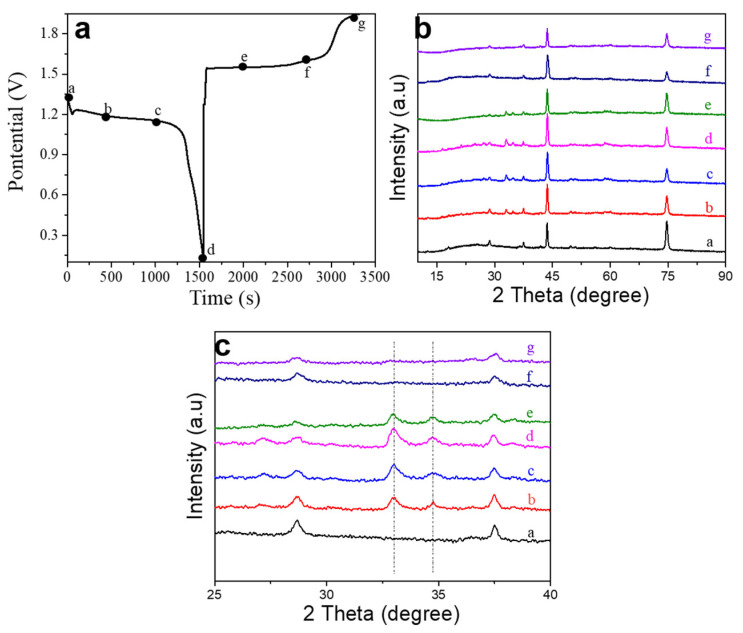
Electrode reaction mechanisms of MnO_2_-1 in ZIBs. (**a**) Charge/discharge curves of the MnO_2_-1 electrode; the dots in the figure indicate that samples at that charge/discharge depth were used for ex situ XRD analysis; (**b**) ex situ XRD patterns of the MnO_2_-1 electrode at various charge/discharge states; (**c**) magnified XRD patterns with 2θ from 25° to 40°.

To further explore the working principle of the MnO_2_-1 electrode, we investigated the ex situ XRD of the materials in different charge and discharge states during the first cycle (as shown in Figure 7b,c). During the discharge process, new peaks at 33° and 34.7°, which are the characteristic peaks of tunnel-type ZnMn_2_O_4_, were observed. During the charge process, the two peaks gradually disappear and return to the initial state. Therefore, the working principle of the MnO_2_-1 electrode during the charge and discharge processes can be expressed as [44]:

MnO_2_ + Zn^2+^ + x × e ⇄ Zn_x_MnO_2_ (0 < x < 1). (1)

## 4. Conclusions

In conclusion, agar was introduced as an EMS for an α-MnO_2_ electrode. After coating with agar, the wettability of the MnO_2_ electrode was greatly improved; this increased the diffusion rate of Zn^2+^ on the surface of the MnO_2_ electrode and reduced the interface impedance. The impedance was the lowest, when the thickness of the coating layer was 4.94 μm (MnO_2_-1), so that the assembled Zn/MnO_2_-1 battery showed excellent rate performance. In addition, because the agar-coating layer promoted the reversibility of the MnO_2_/Mn^2+^ reaction, the Zn/MnO_2_-1 battery showed the highest specific discharge capacity of 384.7 mAh g^−1^ at a current density of 0.25 A g^−1^. Moreover, the agar EMS acted as a colloidal physical barrier on the surface, inhibited the dissolution of Mn^2+^ and maintained the stability of the structure of α-MnO_2_, so that the Zn/MnO_2_-1 battery had excellent cycling stability with a high capacity retention of 85.6% after 500 cycles at a current density of 0.5 A g^−1^.

## Figures and Tables

**Table 1:** Electrochemical performance of the MnO_2_–agar cathode compared with those of different manganese oxide cathodes in recent studies.

Cathode	Anode	Electrolyte	Electrolyte Voltage (V)	Capacity (mAh g^−1^)	Retention/Cycles	Ref.
CNT@MnO_2_	zinc foil	2 M ZnSO_4_/0.2 M MnSO_4_	1–1.85	105.6 mAh g^−1^ (3 mA cm^−2^)	85.72%/1000 cycles	[33]
α-MnO_2_-TiN/TiO	zinc foil	1 M Zn(OAc)/31 M KOAc	0.8–2.0	304.6 mAh·g^−1^ (100 mA g^−1^)	79.7%/600 cycles	[34]
MnCP-X	zinc foil	1 M Zn(CF_3_SO_3_)_2_	0.8–2.0	151 mAh g^−1^ (3 A g^−1^)	89%/500 cycles	[35]
MnO_2_/graphite	zinc foil	2 M ZnSO_4_/0.5 M MnSO_4_	0.8–1.8	80 mAh g^−1^ (1 A g^−1^)	80.8%/1000 cycles	[36]
LPC/δ-MnO_2_	zinc foil	2 M ZnSO_4_/0.2 M MnSO_4_	1.0–1.85	196.1 mAh g^–1^ (5 A g^–1^)	82%/1000 cycles	[37]
MnO_2_-CNTs/CNHs	zinc foil	2 M ZnSO_4_/0.1 M MnSO_4_	1.0–1.9	168.1 mAh g^−1^ (3 A g^−1^)	96.5%/500 cycles	[38]
λ-MnO_2_	zinc foil	1 M Li_2_SO_4_/1 M ZnSO_4_	1.5–2.1	128 mAh g^−1^ (2 C)	83%/1,000 cycles	[39]
MnO_2_/GO	zinc foil	2 M ZnSO_4_/0.2 M MnSO_4_	0.9–1.8	190mAh g^−1^ (3C)	91%/cycles	[40]
MnO_2_/carbon	zinc foil	2 M ZnSO_4_/0.2 M MnSO_4_	0.9–1.8	240 mAh g^−1^ (0.1 A g^−1^)	58.33%/300 cycles	[41]
MnO_2_/rGO/PANI	zinc foil	2 M ZnSO_4_	0.8–1.8	100.6 mAh·g^−1^ (1 A g^−1^)	82.7%/600 cycles	[42]
MnO_2_/OLC	zinc foil	1 M ZnSO_4_/0.1M MnSO_4_	1.0–1.8	168 mAh g^−1^ (246 mA g^−1^)	93%/100 cycles	[43]
MnO_2_–agar	zinc foil	2 M ZnSO_4_/0.5 M MnSO_4_	0.1–1.95	260.6 mAh g^−1^ (0.5 A g^−1^)	95.4%/500 cycles	This work

## Data Availability

All the data are available within the manuscript.

## Supplementary Materials

The following are available online at https://www.mdpi.com/article/10.3390/ma14174895/s1, Figure S1: FTIR spectrum of agar/MnO_2_, Figure S2: Fitting and calculation of slope in the EIS low frequency region of three batteries, Figure S3: Electrochemical charge–discharge curves at different current densities over the potential range 0.1–1.95 V: (a) MnO_2_, (b) MnO_2_-2, Figure S4: Electrochemical charge–discharge curves at a current density of 0.5 A g^−1^ over the potential range 0.1–1.95 V: (a) MnO_2_, (b) MnO_2_-2.
