# Supplementary material for: Agar Acts as Cathode Microskin to Extend the Cycling Life of Zn//α-MnO2 Batteries
Source: Materials (Basel). 2021 Aug 27;14(17):4895. doi: 10.3390/ma14174895 (PMC8432668; doi:10.3390/ma14174895)
Supplement: Supplementary file 1 [file materials-14-04895-s001.zip › materials-1320889-supplementary.pdf]

materials

MDPI

Supplementary Materials

Agar Acts as Cathode Microskin to Extend the Cycling Life of Zn//α-MnO_2 Batteries

Linqing Zuo [1], Haodong Sun [1], Xinhai Yuan [1], Juan Wen [1], Xi Chen [1], Shiyu Zhou [1], Yuping Wu [1,*] and Teunis van Ree [2]

[1] China State Key Laboratory of Materials-Oriented Chemical Engineering, Institute of Advanced Materials (IAM) & School of Energy Science and Engineering, Nanjing Tech University, Nanjing 210009, China; 201861122095@njtech.edu.cn (L.Z.); Sun-Haodong@njtech.edu.cn (H.S.); 15026765265@163.com (X.Y.); wenjuan@njtech.edu.cn (J.W.); chenxi@njtech.edu.cn (X.C.); 201961108012@njtech.edu.cn (S.Z.)

[2] Department of Chemistry, University of Venda, Thohoyandou 0950, South Africa; Teuns.VanRee@univen.ac.za

* Correspondence: wuyp@fudan.edu.cn

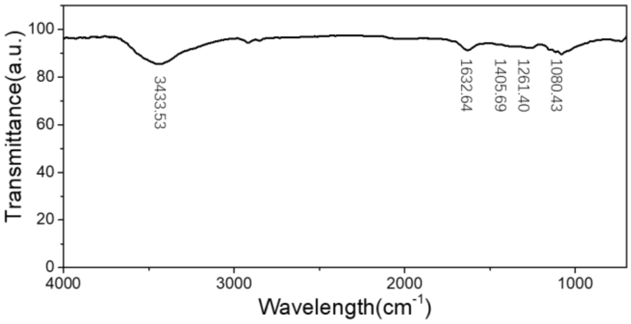

Figure S1. FTIR spectrum of agar/MnO_2.

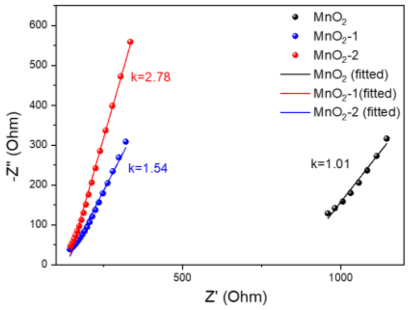

Figure S2. Fitting and calculation of slope in the EIS low frequency region of three batteries.

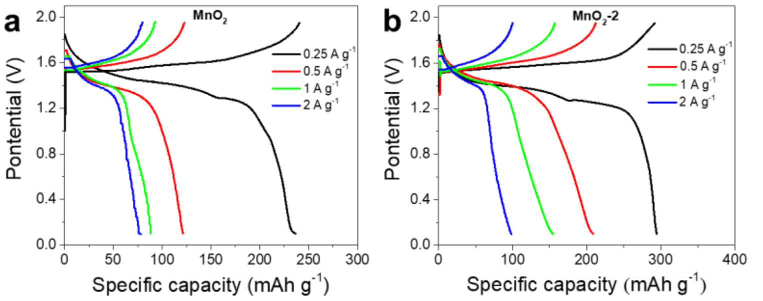

Figure S3. Electrochemical charge–discharge curves at different current densities over the potential range 0.1–1.95 V: (**a**) MnO_2, (**b**) MnO_2-2.

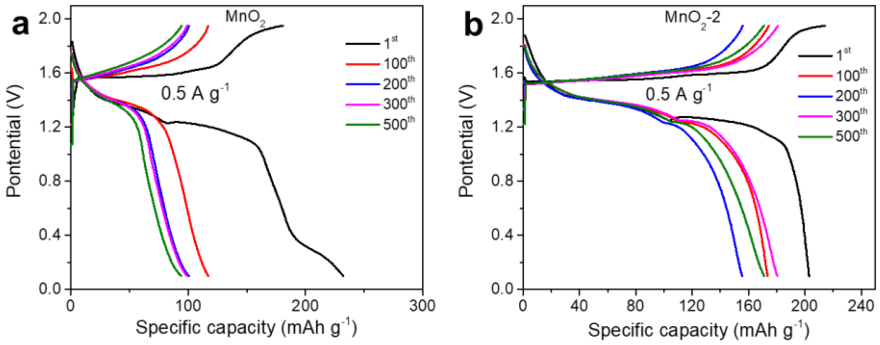

Figure S4. Electrochemical charge–discharge curves at a current density of 0.5 A g^{-1} over the potential range 0.1–1.95 V: (**a**) MnO_2, (**b**) MnO_2-2.
